# Factors associated with late presentation of suspected tuberculosis cases to tuberculosis management facilities: The case in Dagoretti district, Nairobi, Kenya

**Published:** 2012-07-31

**Authors:** Irene Wambui Njau, Simon Muturi Karanja, Peter Wanzala, Jared Odhiambo Omolo

**Affiliations:** 1 Jomo Kenyatta University of Agriculture and Technology, Nairobi, Kenya; 2 Kenya Medical Research Institute, Nairobi, Kenya; 3 Field Epidemiology and Laboratory Training Programme, Nairobi, Kenya

**Keywords:** Tuberculosis, late presentation, management, suspects, participants, respondents, Kenya

## Abstract

**Background:**

Tuberculosis is a highly contagious disease accounting for a high number of deaths in the developing countries; its control can be effectively achieved if individuals with the disease receive adequate and timely treatment. The objective of this study was to determine the factors associated with late presentation of suspects to tuberculosis management facilities in Dagoretti district in Nairobi, Kenya.

**Method:**

A cross sectional study was conducted on patients aged 18 years and above attending TB clinics in Dagoretti District, Nairobi Kenya. A total of 426 TB suspects were interviewed. The study covered 8 clinics in Dagoretti district. Analysis was done using SPSS version 16.0 and Epi info version 6, this included Chi Square for Bivariate analysis and Binary Logistic Regression for Multivariate Analysis.

**Results:**

Out of the 426 tuberculosis suspects, 248 (58.2%) suspects had delayed in seeking medical care. In Bivariate analysis male gender (P = 0.039, O.R = 1.51; 95% Confidence Interval; 1.00- 2.27), level of education (Primary class 5-8) (P = 0.001, O.R= 2.06; 95% C.I 1.34-3.19) and place of first medical care (drug store) (P= 0.013, O.R = 1.63; 95% C.I 1.09-2.46) were all significantly associated with late presentation. After multivariate logistic regression, gender (P = 0,019, OR = 1.6), level of education (p = 0.029, OR = 1.26) and place of first medical care (P= 0.01 OR = 1.27), were found to be significantly associated with late presentation.

**Conclusion:**

This study shows that age, level of education and place of first medical care are the factors associated with late presentation of suspects to tuberculosis management facilities.

## Background

Tuberculosis (TB) is a major public health problem in developing countries. Despite intensified global efforts, the number of cases of TB worldwide is increasing. The WHO also estimates that there are nearly 2 million deaths from tuberculosis annually; thus, the disease ranks second only to human immunodeficiency virus (HIV) infection as an infectious cause of death [1]. Patient′s alertness to the symptoms of tuberculosis combined with health workers′ readiness to diagnose the disease and understanding which factors influence this delay is crucial for controlling the spread of the infection within a community [2].

The estimates of the global burden of disease caused by TB in 2009 show that there were 9.4 million incident cases, 14 million prevalent cases, 1.3 million deaths among HIV-negative people and 0.38 million deaths among HIV-positive people. An estimated 11-13% of incident cases were HIV-positive; the African region accounted for approximately 80% of these cases [3, 4]. This means that the disease is still a major problem in the African region. According to the Division of Leprosy, Tuberculosis and Lung Diseases (DLTLD) in Kenya, the country is one of the 22 high TB burden countries in the world which collectively contribute 80% of the global TB disease burden [5].

Early diagnosis and prompt effective therapy form the key elements of TB control. Late presentation of patients to health facilities results to delayed diagnosis. Delay in diagnosis results in increased infectivity in the community and it is estimated that an untreated smear positive patient can infect on average 10 contacts annually and 20 during the natural history of the disease until death [6]. Late presentation to health facilities is a major problem contributing to the high burden and transmission of tuberculosis (TB) in most developing countries: where fewer than half the estimated sputum smear positive pulmonary tuberculosis [3]. It is therefore important to identify the factors that cause patients to present late to health facilities as this will enable policy makers to come up with effective interventions to reduce the delay. Reduction of the time between onsets of TB symptoms to diagnosis is therefore a prerequisite to bring TB epidemic under control [7].

In Kenya, there are few studies which have been done to establish the factors that cause patients to delay in seeking medical care. Published data about factors causing late presentation of patients to TB management facilities in Nairobi is scanty. A study similar to this was done in a rural setting in the Western part of the country and it attributed a substantial part of the total delay (95%) to late presentation of patients in seeking medical care after onset of symptoms [8]. Effective control of TB can only be achieved if factors causing delay are established and ways of eliminating such factors ascertained. The purpose of this study was to establish the factors that lead to patients presenting with TB like symptoms delaying to seek medical care.

## Methods

### Study setting and population

This study was conducted in Dagoretti district in Nairobi Kenya. The District had a case notification rate of 585.2 per 100,000 populations as per 2009 data from DLTLD. According to 1999 census the district had a total population of 240,081. The district has a total of 26 TB facilities, however only eight facilities had functional laboratories for conducting TB diagnosis, the other facilities were treatment only sites. The eight facilities were the ones covered in this study. The facilities covered included: Kenyatta National hospital, Mbagathi district hospital, Riruta health center, Waithaka health center, Chandaria community hospital, Mid-hill hospital, Mary Mission hospital and Mutuini district hospital. The study only covered pulmonary TB suspects.

### Study design

A cross sectional study design was used in this study. TB suspects in these facilities were interviewed and followed up for the final diagnosis from the facilities. The study was conducted from February 2011 to July 2011.

### Ethical issues

The study was performed according to the modified Helsinki Declaration (WHO, 2001). Permission to perform the study was obtained from Kenya Medical Research (KEMRI) Ethical Research Committee, Division of Leprosy, Tuberculosis and Lung Diseases (DLTLD) so as to access the facilities. Permission to conduct the study in Kenyatta National Hospital (KNH) was obtained from the KNH ethical clearance committee. Permission was also obtained from the Provincial and District coordinator in the DLTLD. Permission was also obtained from the management of the facilities that were covered.

### Measurements

The dependent variable in this study was late presentation which was defined as presenting to a facility after three weeks from onset of symptoms. The independent variables captured in the questionnaire included; Age, Sex, Level of education, Knowledge about TB, Employment status, Cost of travel to health facilities, Distance to clinics, Marital status, Level of income, Alcohol intake and smoking, Alternative medical care e.g. herbalist and HIV/AIDS status. The duration that the TB suspects took to visit a health facility from the onset of symptoms was recorded.

### Data management and analysis

Data from the questionnaires was entered to an access database and imported to Ms excel where data cleaning, coding and validation was done. The data was then transferred to both SPSS version 16 and Epi Info version 6 for analysis. Analysis of descriptive statistics was done, bivariate analysis was done using chi square which generated P-values. A significant level of P

## Results

### Participants age and gender analysis

A total of 426 TB suspects were interviewed. The number of male participants were 245 (57.5%), while female were 181 (42.5%). Analysis of the age categories of the participants showed that majority of the participants were aged between 30-39 years (40.8%).

### Marital status, Employment status and level of education of the participants

Most of the respondents were married (64.6%) compared to those who were single or divorced. With regard to employment status, most of the respondents were casual laborers (46.2%). The level of education analysis revealed that most of the respondents had attained education up to primary class 5-8 Kenyan system.

### Knowledge about TB and reasons for late presentation

Majority of the participants thought that TB is transmitted through TB patients coughing directly to others (54.5%). However other respondents thought it was transmitted through other means for example sexual intercourse while others did not know the mode of transmission. 83.1% of the respondents knew that TB has a cure while 41.1% of the respondents knew that there is a relationship between TB and HIV. When asked why they were late to seek medical care after onset of symptoms, majority of the respondents that they thought the symptoms were not serious and would go away (63.8%). Other selected factors that were analyzed are shown in Table 1 and Table 2. Analysis of the various symptoms that the participants presented with is shown in Table 3.

**Table 1:** Selected characteristics of the respondents interviewed

Variable/ Category	n	95% Confidence Interval		
		Lower limit	Upper Limit	Proportion
Age (Years)				
18-20	19	2.7	6.9	4.5
21-29	121	24.2	32.9	28.4
30-39	174	36.1	45.7	40.8
40-49	81	15.4	23.1	19
50-59	21	3.1	7.4	4.9
60 +	10	1.1	4.3	2.3
**Marital Status**				
Single	84	16.04	23.8	19.7
Married	275	59.8	69.1	64.6
Separated/divorced	39	6.6	12.3	9.2
Widow/widower	28	4.4	9.4	6.6
**Employment status**				
Permanent	45	7.8	13.9	10.6
Casual	197	41.4	51.1	46.2
Unemployed	32	5.2	10.4	7.5
Business	125	25.1	33.9	29.3
Other (Retired or Student)	27	4.2	9.1	6.3
**Respondent's reasons for delay**				
Thought symptoms were mild	272	59.1	68.4	63.8
Not sure where to seek care	20	2.9	7.2	4.7
Lacked transport	23	3.5	8	5.4
Self-medication	51	9	15.4	12
Fear it could be TB	26	4	8.8	6.1
Others	34	5.6	11	8
**Respondents place of first medical care after onset of symptoms**				
Over the counter drugs	217	46.1	55.8	50.9
Herbalist	25	3.8	8.5	5.9
Government of Kenya facilities	81	15.4	23.1	19
Mission/Private	84	16	23.8	19.7
Others	19	2.7	6.9	4.5

**Table 2:** Selected characteristics of the respondents interviewed

Variable/ Category	n	95% Confidence Interval		
		Lower limit	Upper Limit	Proportion
Number of rooms				
1 room	247	53.1	62.7	58
2-3 rooms	142	28.9	38	33.3
More than 3 rooms	37	11.8	11.8	8.7
**Household size**				
1 to 3	227	48.4	58.1	53.3
4 to 6	161	33.1	42.6	37.8
More than 6	38	6.4	12.04	8.9
**Level of income (Kshs)**				
Below 3000	67	12.4	19.5	15.7
3001-6000	104	20.4	28.8	24.4
6001-9000	84	16.04	23.8	19.7
Above 9000	132	26.6	35.6	31
None	39	6.6	12.3	9.2
**Mode of transport**				
Foot	151	30.9	40.2	35.4
Bus/Matatu	262	56.7	66.1	61.5
Own vehicle	13	1.6	5.2	3.1
**Distance to facility**				
Less than 1km	194	40.7	50.4	45.5
1-2 km	161	33.2	42.6	37.8
More than 2 km	71	13.3	20.6	16.7
**Cost of visit to health facilities (Kshs)**				
Less than 100	359	80.5	87.6	84.3
100-200	24	3.6	8.3	5.6
more than 300	43	7.4	13.4	10.1

**Table 3:** Symptoms reported in the respondents

Symptom	n	%	95% C.I
Cough	400	93.9	(91.2-96.0)
Cough with blood	119	27.9	(23.7-32.5)
Difficulty in breathing	211	49.5	(44.7-54.4)
Chest pains	260	61	(56.2-65.7)
Fever and Night sweats	243	57	(52.2-61.8)
Loss of weight	166	39	(34.3-43.8)

### Bivariate analysis of the factors associated with late presentation to health facilities

In reference to late presentation, 248 (58.2%) participants presented late to the health facilities as compared to 178 (41.8%) who presented within three weeks after onset of symptoms.

Gender was significantly associated with late presentation (P = 0.039 X^2^=4.248), with more men presenting late than women. The level of education had significant association with late presentation (P = 0.001, X^2^= 19.460). Individuals who had attained education up to primary class 5 to 8 had the highest number of late presentation (44%).

The place where the respondents sought medical care first after onset of symptoms was significantly associated with late presentation (P = 0.001, X^2^=18.531). Most of the respondents who presented late had sought care from drug stores after onset of symptoms. Bivariate analysis of other factors that were not significant is shown in Table 4 and Table 5.

**Table 4:** Bivariate analysis of the factors associated with late presentation to health facilities

Variable/category	Late		Early		O. R	95% C.I for O. R		P value
	n	%	n	%		Lower	Upper	
Age in years								
30-39 *	114	46	60	33.7	1			
18-20	10	4	9	5.1	1.71	0.6	4.87	0.389
21-29	63	25.4	58	32.6	1.75	1.06	2.89	**0.028**
40-49	43	17.3	38	21.3	1.68	0.95	2.98	0.078
50-59	12	4.8	9	5.1	1.42	0.52	3.89	0.605
60 +	6	2.4	4	2.2	1.27	0.29	5.33	0.741
**Marital Status**								
Married*	158	63.7	117	65.7	1			
Single	48	19.4	36	20.2	1.01	0.6	1.71	0.94
Divorced	25	10.1	14	7.9	0.76	0.36	1.59	0.539
Widowed	17	6.9	11	6.2	0.87	0.37	2.06	0.895
**Employment status**								
Casual*	118	47.6	79	44.4	1			
Permanent	23	9.3	22	12.4	1.43	0.71	2.87	0.362
Unemployed	22	8.9	10	5.6	0.68	0.28	1.61	0.449
Business	70	28.2	55	30.9	1.17	0.73	1.9	0.564
Other	15	6	12	6.7	1.19	0.49	2.88	0.824
**Reasons for delay**								
Thought symptoms would go away*	165	66.5	107	60.1	1			
Not sure where to seek medical care	9	3.6	11	6.2	1.88	0.7	5.14	0.254
Lacked transport	13	5.2	10	5.6	1.19	0.46	3.01	0.867
Self-medication	27	10.9	24	13.5	1.37	0.72	2.61	0.382
Fear it could be TB	16	6.5	10	5.6	0.96	0.39	2.35	0.902
Others	18	7.3	16	9	1.37	0.63	2.97	0.496
**Alcohol intake**								
No*	173	69.8	129	72.5	1			
Yes	75	30.2	49	27.5	0.88	0.56	1.37	0.617
**Smoking**								
No *	187	75.4	146	82	1			
Yes	61	24.6	32	18	0.67	0.4	1.11	0.13

* Reference categories

**Table 5:** Bivariate analysis of selected factors in relation to late presentation

Variable/category	Late		Early		OR	95% C.I for O. R		P value
	n	%	n	%		Lower	Upper	
Monthly income								
*Above 9000	69	27.8	63	35.4	1			
0-3000	60	24.2	46	25.8	0.84	0.49	1.45	0.592
3001-6000	67	27	37	20.8	0.6	0.34	1.06	0.081
6001-9000	52	21	32	18	0.67	0.37	1.22	0.211
**Household size**								
*1 to 3	126	50.8	101	56.7	1			
4 to 6	95	38.3	66	37.1	0.87	0.56	1.33	0.561
> 6	27	10.9	11	6.2	0.51	0.22	1.13	0.106
**Number of rooms**								
*1	143	57.7	104	58.4	1			
2 to 3	86	34.7	56	31.5	0.9	0.57	1.37	0.683
>3	19	7.7	18	10.1	1.3	0.62	2.75	0.567
**Mode of transport**								
*By foot	154	62.1	107	60.1	1			
Bus/matatu	87	35.1	64	36	1.06	0.69	1.62	0.864
Own vehicle	6	2.4	7	3.9	1.68	0.49	5.82	0.529
Others	1	0.4	0	0	0	0	25.26	1
**Distance**								
*< 1km	107	43.1	87	48.9	1			
1-2 km	95	38.3	66	37.1	0.85	0.55	1.33	0.534
>2km	46	18.5	25	14	0.67	0.37	1.22	0.206
**Cost of visit**								
* < 100 shs	202	81.5	157	88.2	1			
100-200 shs	18	7.3	6	3.4	0.43	0.15	1.18	0.113
>200 shs	28	11.3	15	8.4	0.69	0.34	1.39	0.345

### Multivariate analysis

Only those variables that were significant at the bivariate level were entered in the binary logistic regression model. Gender, Place of first medical care after onset of symptoms and level of education were all significant at multivariate level. An analysis of these variables is indicated in Table 6.

**Table 6:** Multivariate analysis

Predictor variables	b	S.E. (b)	df	P value	Adjusted	95.0% C.I. for odds ratio	
					odds ratio	Lower	Upper
Age in years	0.018	0.1	1	0.855	1.019	0.837	1.24
Gender	0.487	0.208	1	0.019	1.628	1.083	2.448
Level of education	0.231	0.106	1	0.03	1.259	1.023	1.55
Place of first medical care	0.239	0.074	1	0.001	1.27	1.098	1.469

## Discussion

This cross-sectional study is the first to document the factors associated with late presentation of suspected TB cases to management facilities in Dagoretti district. Interventions in the control of TB should be aimed at reducing the time between onset of symptoms and diagnosis. Reduction of this period in turn reduces the period of infectivity within the community.

More men than women were interviewed in this study. Gender was a significant factor in this study with more men (61.7%) presenting late to health facilities than women (P = 0.039). Previous studies have reported similar findings; a study in South Africa [9] and Uganda [10], reported that male gender is associated with longer patient delay. Since previous studies female are better health seekers than male [11], it means that men are likely to present to health facilities after the disease has already progressed. Women are most likely to present themselves frequently to medical facilities as compared to men due to issues regarding reproductive health or when they are taking their children to the hospitals and therefore their symptoms are more likely to be captured and treated on time.

Age was not a contributing factor in this study; however age group 30-39 years apparently had the highest proportion of late presentation (46%). Majority of the respondents who presented late in this age group were male (62.3%). The delays in this group could be because the respondents are most likely involved in so many other responsibilities that the symptoms go on unnoticed. For instance they are likely to be involved in work or could be busy looking after their families. They are therefore likely to seek care when the symptoms have already progressed. In other studies different age groups have been attributed to patient delay. A similar study in Western Kenya found that most delays occurred in age group 45 years and over [8]

The level of education of the participants of this study was low with majority of them having attained education up to primary class 5-8. There was significant association between the level of education and late presentation of the suspected TB cases to management facilities (P = 0.001). This was in line with a study in Western Ethiopia (P = 0.074) [12]. Education would provide individuals on knowledge on the importance of timely diagnosis and the importance of seeking proper medical care after falling ill. This means that individuals with low levels of education are less likely to know the importance of seeking medical care as soon as symptoms start. They may not able to know what disease the symptoms are associated with and also the symptoms may also seem too mild to warrant any medical attention.

The first place where the participants sought medical care (drug store) after onset of symptoms was associated with late presentation in this study (P = 0.001). Majority of the respondents revealed that they bought over the counter drugs after onset of the symptoms (56%). This study shows that majority of the patients sought medical care after other ways of relieving the symptoms had failed or the symptoms had worsened. The first place of medical care has been seen as a contributing factor to late presentation in several studies. For example a study in Kampala showed that 78% of the participants sought care first in drug stores after onset of symptoms [10] TB symptoms are not so specific and can be easily confused with symptoms of other diseases for example Common cold. Patients would most likely deem the symptoms as mild and opt to look for other ways of relieving the symptoms before seeking proper medical care.

The participants had varying reasons for presenting late to the management facilities. However majority of the respondents thought that the symptoms were mild and would go away (63.8%). The symptoms could easily be confused with a common cold and therefore individuals are less likely to seek medical care in time. In a study in Southern India, the participants revealed that they did not think the symptoms were serious enough to warrant medical care [13] In this study, most of the delays were noted among those respondents who thought that their symptoms were mild (66.5%).

Several limitations were observed in this study. Information for this study relied on the respondents responses and therefore was prone to recall bias. The participants were most likely unable to reveal if they visited herbalists for treatment due to fear of criticism. The study dealt solely on patient interviews and was therefore unable to establish health system factors that lead to delay in diagnosis treatment which is an equally important area.

Despite the limitations, the information obtained could be generalized to the entire Dagoretti district. This study is very important since it is one of the first in Nairobi to publish on the factors that lead to delay. The findings of this study identified the factors that lead to late presentation and recommends that the DLTLD should embark on countrywide campaigns to educate the population on the importance of timely diagnosis and treatment of TB.

## Conclusion

The factors established to lead to patient delay in this study included gender, level of education and the place where medical care was sought first after onset of symptoms. This study recommends that the DLTLD embark on comprehensive campaigns to educate people on the signs and symptoms of TB as well as the importance of timely diagnosis of the disease. More studies on delayed diagnosis should also be done in order to establish factors that were not covered in this study for example the issue of stigmatization.
